# Comparison of the safety of outpatient cervical disc replacement with inpatient cervical disc replacement: A protocol for a meta-analysis

**DOI:** 10.1097/MD.0000000000021609

**Published:** 2020-08-28

**Authors:** Xiaofei Wang, Yang Meng, Hao Liu, Ying Hong, Beiyu Wang

**Affiliations:** a Department of Orthopedic Surgery, West China Hospital, Sichuan University, Sichuan, China; b Department of Anesthesia and Operation Center, West China Hospital, Sichuan University, Sichuan, China; c West China School of Nursing, Sichuan University, Sichuan, China.

**Keywords:** cervical disc replacement, complication, meta-analysis, outpatients, protocol

## Abstract

**Background:**

Cervical disc replacement (CDR) has been widely used as an effective treatment for cervical degenerative disc diseases in recent years. However, the cost of this procedure is very high and may bring a great economic burden to patients and the health care system. It is reported that outpatient procedures can reduce nearly 30% of the costs associated with hospitalization compared with inpatient procedures. However, the safety profile surrounding outpatient CDR remains poorly resolved. This study aims to evaluate the current evidence on the safety of outpatient CDR

**Methods:**

Four English databases were searched. The inclusion and exclusion criteria were developed according to the PICOS principle. The titles and abstracts of the records will be screened by 2 authors independently. Records that meet the eligibility criteria will be screened for a second time by reading the full text. An extraction form will be established for data extraction. Risk of bias assessment will be performed by 2 authors independently using Cochrane risk of bias tool or Newcastle–Ottawa scale. Data synthesis will be conducted using Stata software. Heterogeneity among studies will be assessed using *I*^2^ test. The funnel plot, Egger regression test, and Begg rank correlation test will be used to examine the publication bias.

**Results:**

The results of this meta-analysis will be published in a peer-review journal.

**Conclusion:**

This will be the first meta-analysis that compares the safety of outpatient CDR with inpatient CDR. Our study will help surgeons fully understand the complications and safety profile surrounding outpatient CDR.

**OSF registration number:**

doi.org/10.17605/OSF.IO/3597Z

## 1 Introduction

With the aging of the population and the change of lifestyle, the prevalence of cervical degenerative disc disease is increasing. Severe cases that cause radiculopathy or myelopathy require surgery to decompress the nerve and spinal cord and relief the symptom. Cervical disc replacement (CDR) is a relatively new technique to treat cervical degenerative disc disease and has been applied to clinical practice for twenty years.^[1]^ Compared with the traditional fusion surgery, CDR aims at preserving the range of motion at the pathological level and restoring the physiological properties of the cervical spine to the most extent.^[2,3]^ Several randomized controlled trials comparing the safety and efficiency of CDR with anterior cervical discectomy and fusion have proven the noninferiority of CDR.^[4–8]^ In addition, studies showed that both 1-level and 2-level CDR is more cost-effective in long-term follow-up.^[9,10]^ Therefore, CDR has been widely used in recent years.^[11]^

Although CDR is efficient in treating cervical degenerative disc disease, the cost of this procedure is very high. Saifi et al^[12]^ reported that the mean hospital cost for CDR was $13,197 between 2005 and 2013. Kumar et al^[13]^ used the MarketScan database and found that the mean cost of CDR was $28,664. Therefore, CDR may bring a great economic burden to patients and the health care system.

Recently, with the advances in surgical techniques, anesthesiology, and perioperative care, many surgeries have been transited to outpatient procedures. The convenience of recovering at home rather than in hospital can generally shorten the recovery time and reduce the patient's stress.^[14,15]^ The outpatient surgery does not need an overnight hospital stay; hence, it can keep the hospital-related cost down and save the patient's time. It is reported that outpatient procedures can reduce nearly 30% of the costs associated with hospitalization compared with inpatient procedures.^[16–18]^ Therefore, outpatient CDR could be used as a strategy to reduce costs.

The number of outpatient CDR procedures shows an increasing trend in recent years.^[19]^ Several studies have reported the noninferiority of outpatient CDR compared with outpatient ACDF or inpatient CDR.^[19–27]^ However, the generalizability of these studies is limited by the retrospective study design, small sample size, and low incidence of CDR-associated complications. Therefore, the safety profile surrounding outpatient CDR remains poorly resolved.

To evaluate the current evidence on the safety of outpatient CDR, we aim to perform a meta-analysis of the published studies. We hope our study will help surgeons fully understand the complications and safety profile surrounding outpatient CDR, and help improve the performance of outpatient CDR.

## 2 Methods and analysis

This study has been approved by the review board of Department of Orthopaedic Surgery, West China Hospital, Sichuan University.

### 2.1 Study registration

This protocol has been registered on Open Science Framework (OSF). The registration number is *doi.org/10.17605/OSF.IO/3597Z*. The Registration information is available at *osf.io/szuy9/*. This study will be conducted following the Preferred Reporting Items for Systematic Review and Meta-Analysis Protocols (PRISMA-P) guidelines.

### 2.2 Inclusion criteria

#### 2.2.1 Types of patients

This study will include patients who received CDR procedure due to cervical degenerative disc disease. There are no restrictions on the number of pathological levels of the cervical spine.

#### 2.2.2 Type of intervention

Studies comparing the safety of outpatient CDR with inpatient CDR will be included. There are no restrictions on the type of artificial disc or the number of operated levels. Studies only reported the outcomes of outpatient CDR, which will also be included to analyze the pooled incidence of complications after outpatient CDR.

#### 2.2.3 Type of outcomes

The primary outcomes are listed as follows: Incidence of overall complication and specific complications (such as wound complication, dysphagia, and implant-related complications, if data are available); Readmission and return to operation room during the follow-up of the study. The secondary outcomes include operating time, and length of stay in the hospital or surgery center. We will select the longest follow-up time to evaluate the outcome in each study.

#### 2.2.4 Type of studies

Considering the majority of published studies on this topic are retrospective studies, we will include both prospective studies and retrospective cohort studies.

### 2.3 Information source and search strategy

We searched the following database from inception to April 15, 2020: PubMed, Embase, Web of Science, and Cochrane Library. Articles wrote in English will be included. The following search keywords were used in all databases: “total disc replacement,” “outpatients,” “ambulatory.” The search strategy is available at osf.io/szuy9/. The example search strategy for PubMed is summarized in Table 1. The reference lists of the eligible studies will be reviewed to identify the potential relevant studies.

**Table 1:**
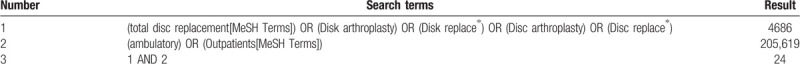
Search strategy for PubMed database.

### 2.4 Study selection

After removing duplicate records, the titles and abstracts of the left records will be screened by 2 authors (XW and HW) independently. Records that meet the eligibility criteria will be screened for a second time by reading the full text. If studies have duplicate data, then the one with a larger sample size will be included. Any disagreement between the 2 authors will be solved by consulting a senior author (HL).

### 2.5 Data extraction

An extraction form will be established for data extraction, which will include the following aspects: Study information, including author name, corresponding author name, affiliation, region, year of publication, journal name, conflict of interest, funds, type of study, inclusion criteria, data source, sample size, type of device, and definition of outpatient surgery; Patient information, including age, gender, body mass index (BMI), smoking and drinking history, physical activity, comorbidities, diagnosis, and pathological level; Surgical information, including operating time, estimated blood loss, surgical level, and length of stay; All reported outcomes, including complications found in outpatient CDR, readmission, and reoperation. Data extraction will be performed by 2 authors (XW and HW), respectively. Any disagreement will be solved by consulting a senior author (HL).

### 2.6 Risk of bias assessment

The Cochrane risk of bias tool will be used to evaluate the quality of randomized controlled trials. The Newcastle–Ottawa scale (NOS) will be used to evaluate the quality of prospective studies and retrospective cohort studies. Two authors (XW and HW) will perform the assessment independently. Any disagreement between the 2 authors will be solved by consulting a senior researcher (HL).

### 2.7 Data synthesis

If quantitative analysis cannot be performed, we will describe the results narratively. If the quantitative analysis is feasible, we will conduct the data analysis using Stata (V.14; StataCorp, College Station, TX) software. Continuous variables will be displayed as mean difference (MD) with 95% confidence interval (95% CI). Categorical variables will be displayed as odds ratio (OR) with 95% CI. The incidence of overall complication of outpatient CDR will be pooled and displayed with 95% CI. The heterogeneity among the included studies will be assessed using the *I*^2^ test. An *I*^2^ value >50% will be considered as high heterogeneity, and data will be analyzed using the random-effect model. Otherwise, the data will be analyzed using the fixed model.

If data are available, we will perform subgroup analyses according to age, country, surgical level, diagnosis, study design, and other confounding factors. Sensitivity analysis will also be performed to evaluate the robustness of our results.

The funnel plot, Egger regression test, and Begg rank correlation test will be used to examine the publication bias.

### 2.8 Quality of evidence

The quality of evidence will be evaluated following the Grading of Recommendations, Assessment, Development, and Evaluation (GRADE) approach according to previous studies. The evaluation will be performed by 2 authors (XW and HW) in pairs. The quality will be classified into high, moderate, low, and very low. Any disagreement between the 2 authors will be solved by consulting a senior researcher (HL).

### 2.9 Ethics and dissemination

This study will not include the patient or public. However, the results of this study will be discussed with surgeons to improve the performance of the CDR procedure. The results will be submitted to a peer-reviewed journal.

## 3 Discussion

This will be the first meta-analysis that compares the safety of outpatient CDR with inpatient CDR. Recently, with the advances of anesthesiology and perioperative care regimen, many surgeries have been transited to the outpatient setting. However, the application of outpatient CDR is still in the beginning phase. Although several studies have proven the noninferiority of outpatient CDR, there is no systematic review and meta-analysis that comprehensively discuss the safety and efficiency of outpatient CDR. We hope our study will help surgeons fully understand the complications and safety profile surrounding outpatient CDR. Considering the number of prospective studies is limited, we will include retrospective cohort studies to improve the quality of this study. Although the retrospective studies may have heterogeneity and inferior quality, we will assess the bias and the overall quality to make our results reliable.

## Author contributions

XW and YM are joint first authors. YM obtained funding. XW designed the study. XW and HW designed the search strategy and planned data extraction. XW drafted the manuscript. YH, BW, and HL reviewed the draft. All authors have read and approved the final manuscript.

**Conceptualization:** Xiaofei Wang.

**Funding acquisition:** Yang Meng.

**Methodology:** Xiaofei Wang.

**Project administration:** Yang Meng, Hao Liu, Ying Hong.

**Resources:** Hao Liu.

**Supervision:** Hao Liu, Ying Hong, Beiyu Wang.

**Writing – original draft:** Xiaofei Wang.

**Writing – review & editing:** Yang Meng, Hao Liu, Ying Hong, Beiyu Wang.

## References

[1] Hallab N Link HD McAfee PC Biomaterial optimization in total disc arthroplasty. Spine (Phila Pa 1976) 2003;28:S139 52. 14560185 10.1097/01.BRS.0000092214.87225.80

[2] Xu S Liang Y Zhu Z Adjacent segment degeneration or disease after cervical total disc replacement: a meta-analysis of randomized controlled trials. J Orthop Surg Res 2018;13:244. 30285807 10.1186/s13018-018-0940-9 PMC6169069

[3] Wang Q-L Tu Z-M Hu P Long-term results comparing cervical disc arthroplasty to anterior cervical discectomy and fusion: a systematic review and meta-analysis of randomized controlled trials. Orthop Surg 2019;12:16 30. 31863642 10.1111/os.12585 PMC7031601

[4] Gornet MF Lanman TH Burkus JK Two-level cervical disc arthroplasty versus anterior cervical discectomy and fusion: 10-year outcomes of a prospective, randomized investigational device exemption clinical trial [published online ahead of print, 2019 Jun 21]. J Neurosurg Spine 2019;1 1. doi: 10.3171/2019.4.SPINE19157. 10.3171/2019.4.SPINE19157 31226684

[5] Gornet MF Burkus JK Shaffrey ME Cervical disc arthroplasty: 10-year outcomes of the Prestige LP cervical disc at a single level. J Neurosurg Spine 2019;31:317 25. 31075769 10.3171/2019.2.SPINE1956

[6] Lavelle WF Riew KD Levi AD Ten-year outcomes of cervical disc replacement with the BRYAN cervical disc: results from a prospective, randomized controlled clinical trial. Spine (Phila Pa 1976) 2019;44:601 8. 30325888 10.1097/BRS.0000000000002907

[7] Mehren C Heider F Siepe CJ Clinical and radiological outcome at 10 years of follow-up after total cervical disc replacement. Eur Spine J 2017;26:2441 9. 28676980 10.1007/s00586-017-5204-6

[8] Yang S-D Zhu Y-B Yan S-Z Anterior cervical discectomy and fusion surgery versus total disc replacement: a comparative study with minimum of 10-year follow-up. Sci Rep 2017;7:16443. 29180636 10.1038/s41598-017-16670-1 PMC5703962

[9] Ament JD Yang Z Nunley P Cost-effectiveness of cervical total disc replacement vs fusion for the treatment of 2-level symptomatic degenerative disc disease. JAMA Surg 2014;149:1231 9. 25321869 10.1001/jamasurg.2014.716

[10] Kim JS Dowdell J Cheung ZB The seven-year cost-effectiveness of anterior cervical discectomy and fusion versus cervical disc arthroplasty. Spine (Phila Pa 1976) 2018;43:1543 51. 29642136 10.1097/BRS.0000000000002665

[11] Jain NS Nguyen A Formanek B Cervical disc replacement: trends, costs, and complications. Asian Spine J 2020;[Epub ahead of print]. 10.31616/asj.2019.0246 PMC7595820 32213792

[12] Saifi C Fein AW Cazzulino A Trends in resource utilization and rate of cervical disc arthroplasty and anterior cervical discectomy and fusion throughout the United States from 2006 to 2013. Spine J 2018;18:1022 9. 29128581 10.1016/j.spinee.2017.10.072

[13] Kumar C Dietz N Sharma M Long-term comparison of health care utilization and reoperation rates in patients undergoing cervical disc arthroplasty and anterior cervical discectomy and fusion for cervical degenerative disc disease. World Neurosurg 2020;134:e855 65. 31733395 10.1016/j.wneu.2019.11.012

[14] Sheperd CS Young WF Instrumented outpatient anterior cervical discectomy and fusion: is it safe? Int Surg 2012;97:86 9. 23102004 10.9738/CC35.1 PMC3723190

[15] Yerneni K Burke JF Chunduru P Safety of outpatient anterior cervical discectomy and fusion: a systematic review and meta-analysis. Neurosurgery 2020;86:30 45. 30690479 10.1093/neuros/nyy636

[16] Wang MC Kreuter W Wolfla CE Trends and variations in cervical spine surgery in the United States: Medicare beneficiaries, 1992 to 2005. Spine (Phila Pa 1976) 2009;34:955 61. discussion 962–963. 19352223 10.1097/BRS.0b013e31819e2fd5

[17] McGirt MJ Godil SS Asher AL Quality analysis of anterior cervical discectomy and fusion in the outpatient versus inpatient setting: analysis of 7288 patients from the NSQIP database. Neurosurg Focus 2015;39:E9. 10.3171/2015.9.FOCUS15335 26621423

[18] Adamson T Godil SS Mehrlich M Anterior cervical discectomy and fusion in the outpatient ambulatory surgery setting compared with the inpatient hospital setting: analysis of 1000 consecutive cases. J Neurosurg Spine 2016;24:878 84. 26849708 10.3171/2015.8.SPINE14284

[19] Hill P Vaishnav A Kushwaha B Comparison of inpatient and outpatient preoperative factors and postoperative outcomes in 2-level cervical disc arthroplasty. Neurospine 2018;15:376 82. 30531659 10.14245/ns.1836102.051 PMC6347354

[20] Bovonratwet P Fu MC Tyagi V Safety of outpatient single-level cervical total disc replacement: a propensity-matched multi-institutional study. Spine (Phila Pa 1976) 2019;44:E530 8. 30247372 10.1097/BRS.0000000000002884

[21] Gornet MF Buttermann GR Wohns R Safety and efficiency of cervical disc arthroplasty in ambulatory surgery centers vs. hospital settings. Int J Spine Surg 2018;12:557 64. 30364904 10.14444/5068 PMC6198634

[22] Segal DN Wilson JM Staley C Outpatient and inpatient single-level cervical total disc replacement. Spine (Phila Pa 1976) 2019;44:79 83. 29894451 10.1097/BRS.0000000000002739

[23] Purger DA Pendharkar AV Ho AL Analysis of outcomes and cost of inpatient and ambulatory anterior cervical disk replacement using a state-level database. Clin Spine Surg 2019;32:E372 9. 31180992 10.1097/BSD.0000000000000840

[24] Chin KR Pencle FJR Seale JA Clinical outcomes of outpatient cervical total disc replacement compared with outpatient anterior cervical discectomy and fusion. Spine (Phila Pa 1976) 2017;42:E567 e574. 27755491 10.1097/BRS.0000000000001936

[25] Cuéllar JM Lanman TH Rasouli A The safety of single and multilevel cervical total disc replacement in ambulatory surgery centers. Spine (Phila Pa 1976) 2020;45:512 21. 31703051 10.1097/BRS.0000000000003307

[26] Chin KR Pencle FJR Coombs AV Safety and outcome of outpatient 2-level hybrid anterior cervical discectomy and fusion plus adjacent total disc replacement. West Indian Med J 2017;66:440 4.

[27] Wohns R Safety and cost-effectiveness of outpatient cervical disc arthroplasty. Surg Neurol Int 2010;1:77. 21206539 10.4103/2152-7806.73803 PMC3011106

